# Population pharmacokinetic modeling of the Qishe pill in three major traditional Chinese medicine-defined constitutional types of healthy Chinese subjects: study protocol for a randomized controlled trial

**DOI:** 10.1186/s13063-015-0568-6

**Published:** 2015-02-26

**Authors:** Yue-li Sun, Ting Hou, Shu-fen Liu, Zhong-liang Zhang, Ning Zhang, Min Yao, Long Yang, Qi Shi, Xue-jun Cui, Yong-jun Wang

**Affiliations:** Longhua Hospital, Shanghai University of Traditional Chinese Medicine, No. 725, South Wanping Road, Shanghai, 200032 P.R. China; Spine Institute, Shanghai University of Traditional Chinese Medicine, No. 725, South Wanping Road, Shanghai, 200032 P.R. China; College of Traditional Chinese Herbal Medicine, Shanghai University of Traditional Chinese Medicine, No. 1200 Cailun Road, Shanghai, 201203 P.R. China; Zhe Jiang Biosia Pharmaceutical Co., Ltd, No.1938 Xinqun Road, Pinghu City, Zhejiang Province P.R. China

**Keywords:** Personalized medicine, Individualized medicine, Traditional Chinese Medicine, Multiomics, Population pharmacokinetics

## Abstract

**Background:**

High incidences of neck pain morbidity are challenging in various situations for populations based on their demographic, physiological and pathological characteristics. Chinese proprietary herbal medicines, as Complementary and Alternative Medicine (CAM) products, are usually developed from well-established and long-standing recipes formulated as tablets or capsules. However, good quantification and strict standardization are still needed for implementation of individualized therapies. The Qishe pill was developed and has been used clinically since 2009. The Qishe pill’s personalized medicine should be documented and administered to various patients according to the ancient *TCM* system, a classification of personalized constitution types, established to determine predisposition and prognosis to diseases as well as therapy and life-style administration. Therefore, we describe the population pharmacokinetic profile of the Qishe pill and compare its metabolic rate in the three major constitution types (*Qi-*Deficiency, *Yin*-Deficiency and *Blood-*Stasis) to address major challenges to individualized standardized TCM.

**Methods/Design:**

Healthy subjects (N = 108) selected based on constitutional types will be assessed, and standardized pharmacokinetic protocol will be used for assessing demographic, physiological, and pathological data. Laboratory biomarkers will be evaluated and blood samples collected for pharmacokinetics(PK) analysis and second-generation gene sequencing. In single-dose administrations, subjects in each constitutional type cohort (N = 36) will be randomly divided into three groups to receive different Qishe pill doses (3.75, 7.5 and 15 grams).

Multiomics, including next generation sequencing, metabolomics, and proteomics, will complement the Qishe pill’s multilevel assessment, with cytochrome P450 genes as targets. In a comparison with the general population, a systematic population pharmacokinetic (PopPK) model for the Qishe pill will be established and verified.

**Trial registration:**

This study is registered at ClinicalTrials.gov, NCT02294448.15 November 2014.

**Electronic supplementary material:**

The online version of this article (doi:10.1186/s13063-015-0568-6) contains supplementary material, which is available to authorized users.

## Background

Neck pain is one of the most common symptoms of cervical spine disease. Many treatments are available to patients and considered standard in clinical practice. They include common conservative strategies such as medication, physical methods, manual treatments, and education of patients. However, current evidence for the management of neck pain reveals that conservative interventions, especially Chinese herbal medicine, have not been studied in enough detail to assess their efficacy or effectiveness adequately [1]. Possible reasons for the failure of current clinical practice to effectively manage neck pain lie in two main domains: first, the most difficult challenge faced is clinical heterogeneity, which appears at many levels, such as in the sample group, type of herbal medicine used and outcomes measured [2]; and second, although Chinese proprietary herbal medicines are usually based on well-established and long-standing recipes and formulated as tablets or capsules for commerce, convenience and/or palatability, good quantification and strict detailed standardization still need to be improved.

As individualized diagnostic and therapeutic strategies have become a trend in modern medicine, they might help improve the efficacy and safety, predicting individual outcome and assessing risk, which make individualized prevention and early intervention strategies conceivable and practicable. According to the ancient *TCM* system, a classification of individualized constitution types has been established to determine predisposition and prognosis to diseases as well as therapy and life-style administration [3]. An official Classification and Determination of TCM Constitution [4] was released by the State Administration of TCM and the constitution branch of the Chinese Medical Association in April, 2009. Meanwhile, a standardized scale called Constitution in Chinese Medicine Questionnaire (CCMQ) [5-7] was designed based on the above classification. However, its reliability and validity has not been verified with comprehensive, large sample size trials. In the clinic, it takes a lot of practice for *TCM* specialists to be really efficient at adopting the four *TCM* examinations (observation, listening and smelling, inquiring, feeling the pulse and palpation) to totally assess the participants and determine their constitutional types based on physical, mental, physiological, and pathological attributes, which are called *Ti Zhi.* There are nine kinds of broad constitutional types with a varying degree of predisposition to different diseases (Table 1). Among these, the three most contrasting constitutional types, *Qi-*Deficiency, *Yin*-Deficiency and *Blood-*Stasis, are the most vulnerable to chronic musculoskeletal diseases [3]. In the realm of modern predictive medicine, efforts are being directed toward capturing disease phenotypes with greater precision for successful identification of markers for prospective disease conditions.

**Table 1:** **General characteristics of the nine traditional Chinese medicine** *(* **TCM) constitutional types** [3,13,14]

	**General characteristics**	**Physical characteristics**	**Psychological characteristics**	**Sign of health**	**Sign of illness**
Gentleness	*Yin-Yang* balanced	Full of vitality and well-proportioned	Mild character	Good cold and hot tolerance, sleep well, good appetite and no fatigue	Less disease
Type A					
*Qi-*deficiency^*^	Insufficiency of primordial *Qi*	Weakened muscle^*^	No desire to speak	Short of breath, listless and low voice and easy to sweat and fatigue	Palpitations and influenza
Type B					
*Yang*-deficiency	More insufficiency of primordial *Qi*	Slightly muscle atrophy	More quiet and introverted	Intolerance of cold, loose stool, clear abundant urine and easy to catch a cold	Diarrhea
Type C					
*Yin*-deficiency^*^	*Yin*-fluid deficiency	Slim body	Quick-tempered	Feverish palms and soles, dry mouth and throat, dry eyes, flushing cheek, and dry skin is usual	Fatigue syndrome, muscle fatigue^*^ and insomnia
Type D					
Phlegm-wetness	Body fluid stasis and phlegm-wetness aggregation	Obese body with accumulate fat around abdomen	Prudent	Feeling body heaviness, chest distress and excessive phlegm, sticky mouth, and sticky sweating in usual	Stroke and Diabetes
Type E					
Wet-heat	Internal accumulation of damp-heat	Slim body	Tend to be irritable	likely to suffer from acne, thirsty, bad breath, bitter taste in the mouth in usual	Constipation, scabies and jaundice
Type F					
Blood-stasis^*^	Poor blood circulation	All body types	Tend to be annoying and forgetful	Frequent pain^*^ and ecchymosis and gloomy complexion	Chronic pain^*^
Type G					
*Qi-*depression	Depression and stagnation of *Qi*	Slim body	Depressive, sensitive and suspicious	Frequently sigh, distention and pain in chest	hypochondria
Type H					
Special diathesis	Special diathesis	All body types	All types	Hypersensitiveness	Asthma and urticaria
Type I					

*The three TCM constitutional types (Qi-deficiency, Yin-deficiency and Blood-stasis) have been selected in this trial.

In the early 1990s, the YQHYTL formula was proposed for neck pain and subsequently underwent further investigation to establish the medicine’s efficacy. Based on the YQHYTL formula, the Qishe pill (Shanghai Sundise Traditional Chinese Medicine Co., Ltd, China), which is composed of processed *Radix Astragali*, *Muscone, Szechuan Lovage Rhizome*, *Radix Stephaniae Tetrandrae*, *Caulis Sinomenii*, and *Calculus Bovis Artifactus* (Table 2), was developed and has been widely used in clinical settings since 2009 [8-11]. The Qishe pill is a thin 0.15 g film-coated pill with a slightly penetrating odor and a mildly bitter flavor. Production and processing of the Qishe pill are described as follows: (1) the volatile oil is extracted from *Szechuan Lovage Rhizome* on standby; (2) the remaining drug decoction from *Szechuan Lovage Rhizome* is mixed with *Radix Astragali,* which has been soaked for 30 min, and simmered in 10 volumes of water, twice, for 2 hours each time; (3) the decoction is then vacuum filtrated to a relative density of 1.24 to 1.26 (70°C), and ethanol is added to the concentrated decoction to bring to a 70% alcohol content; (4) after filtration and concentration (ethanol recovery), the remaining sample is vacuum dried and then crushed into a fine powder; (5) from this mixture, which contains 14 volumes of 70% ethanol, *Radix Stephaniae Tetrandrae* and *Caulis Sinomenii* are extracted with the circumfluent alcohol, three times for 2 hours, concentrated with recovery of ethanol, vacuum dried again, and crushed into a fine powder; (6) the volatile oil of *Szechuan Lovage Rhizome* is then mixed with four parts β-cyclodextrin and dried under vacuum; and (7) the porphyrized powder of the *Calculus Bovis Artifactus* and *Muscone* are mixed with the β-cyclodextrin and the other two fine powders mentioned above, pelleted and coated, to obtain the pills called the Qishe pill (drug name).

**Table 2:** Standard formula (capsule ingredients)*

**Chinese Name**	**Pharmaceutical Name**	**Powered Herb, %**
Huang Qi	*Radix Astragali*	28% (13g)
She Xiang	*Muscone*	0. (0.03g)
Chuan Xiong	*Szechuan Lovage Rhizome*	26% (12g)
Fang Ji	*Radix Stephaniae Tetrandrae*	19% (9g)
Qing Feng Teng	*Caulis Sinomenii*	26% (12g)
Niu Huang	*Calculus Bovis Artifactus*	0. (0.3g)

*Pharmaceutical terminology from Hsu [12].

With the high incidence of neck pain morbidity [13-16], and the wide variety of population properties (age, gender, weight, constitutional type, history of disease and life style, etc.) involved, providing an individual patient with optimal treatment at a given time is challenging. Therefore, personalized administration of the Qishe pill should be documented and practiced with multiple patients. Thus, a comprehensive research concerning the population pharmacokinetics (PopPK) of the Qishe pill in Chinese subjects should be conducted. In this study, we describe the population pharmacokinetic profile of the Qishe pill and compare its extent of metabolism in the three major constitution types (*Qi-*Deficiency, *Yin*-Deficiency and *Blood*-Stasis) to address major challenges of individualized and standardized Traditional Chinese Medicine into clinical practice, as a pilot trial.

## Methods/Design

### Study design

This single-center, three cohort, open-label, double-Latin-sequence, crossover study will be conducted in 108 healthy adult subjects (36 subjects in each cohort) who meet the inclusion and exclusion criteria. The experiments will be conducted in accordance with good clinical practice procedures, all applicable regulatory requirements, and the guiding principles of the Declaration of Helsinki. This study was approved by the ethics committee of the institutional review board in Longhua Hospital, Shanghai University of TCM (2013-LCSY-064). After completing baseline description of the study to the enrolled subjects, written informed consent will be obtained from each of them.

The sample size was established according to GCP guidelines of SFDA, which recommend 8 to 12 subjects in a clinical pharmacokinetic trial. The study will take place at the phase I unit of Longhua Hospital, Shanghai University of TCM from Nov. 2014 to Dec 2016.

### Study population

#### Inclusion and exclusion criteria

We will recruit three main cohorts with confirmed *TCM* constitutional types: *Qi-*Deficiency (n = 36), *Yin*-Deficiency (n = 36) and *Blood-*Stasis (n = 36). All subjects will be 20 to 35 years old and with normal weight (18.5 kg/m^2^ ≤BMI <23.0 kg/m^2^). The detailed inclusion and exclusion criteria are presented in Table 3.

**Table 3:** **Inclusion and exclusion criteria**

	**Criteria**
Inclusion	- Aged 20 to 35
	- 18.5 kg/m^2^ ≤body mass index (BMI) <23 kg/m^2^
	- Traditional Chinese medicine (*TCM)-*constitutionally typed as one of the three major types
Exclusion	- History of impaired fasting glucose or diabetes mellitus (past history of diabetes or fasting blood glucose at screening ≥100 mg/dl)
	- History of liver disease (hepatitis, hepatic cirrhosis) or hepatic dysfunction (AST or ALT at screening ≥40 U/L)
	- History of renal dysfunction (creatinine at screening ≥1.2 mg/dl)
	- History of heart disease (heart failure, angina pectoris, myocardial infarction, arrhythmia)
	- History of malignant tumor
	- Having digestive disorders that can interfere with normal absorption of standard diet (gastritis, gastric ulcer, duodenitis, duodenal ulcer, etc.)
	- Smoking during the recent 3 months
	- Alcohol consumption three or more times a week during the recent 3 months
	- Women who were pregnant, intended to become pregnant, or breast- feeding
	- Medicated during the recent month for therapeutic or prophylactic purposes
	- Participating in another clinical trial

All subjects will be surveyed beforehand for their demographic characteristics and lifestyle patterns. The screening will also include blood tests and the Constitution in Chinese Medicine Questionnaire (CCMQ). After exclusion of noneligible participants, the remaining subjects will be assigned identification codes.

### Determination of TCM constitution

According to Classification and Determination of TCM Constitution [4] released by the State Administration of TCM and the constitution branch of the Chinese Medical Association, two TCM specialists will adopt four *TCM* examinations (observation, listening and smelling, inquiring, feeling the pulse and palpation) to totally assess the participants and determine their constitutional types based on physical, mental, physiological, and pathological attributes.

The *TCM* specialists involved are qualified traditional Chinese medical doctors licensed by the Chinese government, with 6 years of traditional Chinese medicine training, minimum 3 years of training in *TCM*, and 9 years of further clinical experience. The constitutional type will be confirmed only when both make concordant decisions. The Constitution in Chinese Medicine Questionnaire (CCMQ) [5-7] will be used to improve objectiveness in decision-making.

### Study procedures

#### Laboratory measurements and clinical assessment

A specific set of laboratory parameters will be measured for every subject on the day of recruitment. These parameters include blood count, electrolyte levels, renal and liver function parameters, blood lipid amounts. Furthermore, age, gender, history of smoking, blood pressure, weight (kg), and height (meters) will be obtained for all subjects. Standard operating procedures have been defined for each clinical examination, including the measurements of blood pressure, height and weight [17].

### Drug administration

Thirty-six healthy adult subjects in each cohort will be randomly divided into three groups to receive three different doses (low, 3.75 g; middle, 7.5 g; or high, 15 g). Subjects will be randomly assigned following simple randomization procedures (computerized random numbers) to each subgroup. Each dose of the Qishe pill (Shanghai Sundise Traditional Chinese Medicine Co., Ltd, China) with the same lot number will be administered orally with water (240 ml) after at least 10-hour fast. The low dose (3.75 mg) was calculated according to the applicable regulatory guidance [18,19], using the No Observable Adverse Effect Level (NOAEL) in the most sensitive species (500 mg/kg/day in Sprague–Dawley rats).

Subjects will be admitted to the study unit the evening before drug administration. On the day of administration, single doses of the Qishe pill will be taken orally, 1h after a standardized meal. Regular standardized meals will be provided 4 h after drug administration. Water is allowed as desired, except for 1 hour before and 2 hours after drug administration. The subjects will remain within a supervised investigational unit for the subsequent 3-d period for pharmacokinetic study and toxicity monitoring, providing 3-day blood and urine samples. Vital signs and clinical laboratory tests were done before and at 24 h after dosing. Furthermore, they will be requested to return to the study unit 4 d after drug administration for a follow-up visit.

### Sample collection

For the PK analysis and second-generation gene sequencing, 5 ml blood samples will be collected into tubes containing ammonium heparinate and centrifuged immediately at 4000 g for 10 min to obtain plasma samples. These samples will be then transferred into polypropylene vials and kept at −20°C until analysis. For the 7.5 and 15 g dose groups, samples will be collected at dosing (time 0), and 15, 30, 45, 60, 90, 120, 150, 180, 240, 360, 480, 600, 720, 1440, 2160 and 2880 min.

Furthermore, a comprehensive set of biomaterials including EDTA-plasma (6 ml), serum (5 ml) and urine (6 ml) will be collected and stored for future laboratory analyses.

### Safety assessments

Safety and tolerability will be assessed subjectively and objectively. Subjective tolerability will be assessed by questioning subjects about any adverse events (AE). Objective tolerability will be assessed at scheduled intervals by vital signs (body temperature, heart rate and blood pressure), electrocardiograms (ECGs), the examination of intravenous infusion sites, and identification of adverse events (AEs). AE information will be collected throughout the study, based on spontaneous volunteer reports, interviews, clinical examinations and laboratory tests (hematology, serum biochemistry, and urinalysis). The investigators will assess all clinical AEs according to the Medical Dictionary for Regular Activities criteria, in terms of intensity (mild, moderate, or severe), duration, outcome and relationship to the study drug. Those which are life threatening, or lead to death, hospitalization, disability, or medical intervention to prevent permanent impairment or damage, will be considered as serious adverse events (SAE). Each adverse event occurring to a subject has to be recorded in the case-report form.

**Figure 1:**
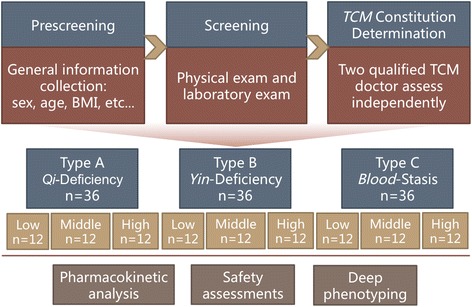
**Patient flowchart detailing the process, including screening, allocation, and analysis.**

All study procedures are illustrated in Figure 1.

### Instrumentation and conditions

The LC-MS/MS system consists of an ULTIMATE 3000 series UHPLC (Thermo Fisher Scientific, USA) coupled to a TSQ QUANTUM ACCESS MAX Mass Spectrometer (Thermo scientific, USA). Chromatographic separation will be performed on a Waters Acquity UPLC BEH C18 column [100 mm*2.1 mm (i.d.); 1.7μm] (Waters, USA). The standards and samples will be separated using a mobile phase consisting of 0.1% formic acid in water (solvent A) and methanol (solvent A). The gradient elution program will be: 0–1 min, 20% A; 1–8 min, 20-100% A; 8–12 min, 100% A; and then back to 20% A to recondition the column for 4 min. The flow rate of the mobile phase will be 0.3 ml/min, with the column temperature maintained at 40°C. ESI ionization performed in the positive ion mode with a spray voltage of 3,000 V and negative mode with a spray voltage of 2500 V will be used. Sheath gas pressure and aux gas pressure will be set at 35 psi and 5 psi, respectively. A capillary temperature of 350°C will be used. Quantification will be performed in the multiple reaction monitoring (MRM) mode. The optimized parameters, such as collision energy (CE) and the tube lens for each compound, are summarized in Table 4. Xcalibur (Ver. 2.1; Thermo scientific, USA) is used to control the UHPLC/TSQ Quantum system, and to acquire and process data.

**Table 4:** **Multiple reaction monitoring (MRM) transitions for quantification of the nine target compounds**

Compound abbreviations*	**Monitoring Mode**	**Parent (m/z)**	**Product (m/z)**	**Tube lens (V)**	**Collision energy (eV)**	**Retention times (min)**
CCS	[M + H]+	285.16	270.30	99	23	6.43
CCSG	[M + H]+	447.24	285.30	98	20	4.89
FAN	[M + H]+	623.44	381.40	130	40	4.89
PAL	[M + H]+	352.23	336.30	89	29	5.44
5-O-M	[M + H]+	453.27	291.30	108	22	6.01
ONO	[M + H]+	431.25	269.30	97	17	6.00
FOR	[M + H]+	269.20	197.30	97	34	7.32
AST-IV	[M + Na]+	807.53	627.40	221	49	8.69
BER	[M + H]+	336.19	320.28	94	30	5.38

*The compound abbreviations and their associated terms:

AST-IV: astragaloside IV; BER: berberine; CCS: calycosin; CCSG: calycosin-7-O-β-D- glucoside; FAN: fangchinoline; FOR: formononetin; ONO: ononin; PAL: palmatine; 5-O-M: 5-O-methylvisammioside.

### Analytical assays

Plasma concentrations and urinary excretion rates of the Qishe pill will be determined for up to 48 h using high-performance liquid chromatography with fluorometric detection according to a method used previously [20].

For sample preparation, plasma samples (100 ml) will be mixed with 100 ml of internal standard (diphenhydramine, purchased from the National Institutes for Food and Drug Control, Beijing, China) working solution. The samples will be extracted with 11:1 methanol–water (600 ml) by vortex-mixing for 1 min at high speed and centrifugation at 12,000xg for 10 min; 20 ml supernatant will be injected in the LC/MS/MS system for analysis.

### Pharmacokinetic parameters

The pharmacokinetic parameters of the Qishe pill will be estimated using noncompartmental methods. The actual blood sampling time will be used, and the maximum plasma concentration (*C*_max_) and time to maximum concentration (*T*_max_) will be recorded. The area under the plasma concentration-time curve (*AUC*) will be calculated using the linear trapezoidal rule. The elimination rate constant (*k*_e_ ) will be estimated from the least-squares regression slope of the terminal plasma concentration. The *AUC* from 0 to infinity (*AUC*_0−∞_) will be calculated by (*AUC*_0→t_ + *C*_t_ /*k*_e_ ) (*C*_t_ is the last plasma concentration measured). The elimination half-life (t_1/2_) will be calculated as ln 2/*k*_e_. The distribution volume (DF) will be calculated by Dose/*AUC*/*k*_e_.

### Deep phenotyping with genomics and functional genomics approaches

Within pharmacokinetics of the Qishe pill, standardized workflows for the omics characterization of biosamples will be developed. Currently, genomic variants will be analyzed for 144 subjects with the aid of the Human- CoreExome + v1.1-Psych Array. For a subset of 144 subjects, whole blood expression data will be generated using Illumina’s HT-12 bead chips. The findings will be complemented with proteomic and metabolomic data using established workflows. Cytochrome P450 genes, such as CYP1A1, CYP1A2, CYP2D6, CYP2C9, CYP2C19, CYP2E1, CYP3A4 and CYP3A5, among other, will be the targets.

Metabolomics provide comprehensive snapshots of the metabolome of body fluids such as plasma or urine. High-throughput metabolomic analyses mainly based on 1H nuclear magnetic resonance (NMR) spectroscopy, a nondestructive analysis with minimal preparation requirements, will be performed. NMR spectroscopy provides robust and reproducible measurements. Mass spectrometry (MS) with high analytical sensitivity will be used for additional detailed studies.

Integrated analyses of these data for associations with clinical and subclinical phenotypes will be performed using the bioinformatics groups of the *TCM* constitutional type classification.

### Medical informatics and data management

The PopPK analysis will be performed with NONMEM [21-23]. The plasma concentration-time profiles for all constituents and derivatives of the Qishe pill will be described by a base structural model using the subroutine ADVAN5 [24]. The PK structural model will be parameterized in terms of apparent clearance and apparent distribution volume (CL/F and V/F), where F is the unknown oral bioavailability; the clearance and distribution volume are CL_M_ and V_M_, respectively. A Bayesian approach conditioned on the population characteristics will be used to estimate specific individual parameters. First-order conditional estimation methods (FOCE) and first-order conditional estimation methods with interaction (FOCE-I) will be tested during model development. The unexplained random variability in individual values of the structural model parameters will be described in the interindividual variability (IIV) model.

**Figure 2:**
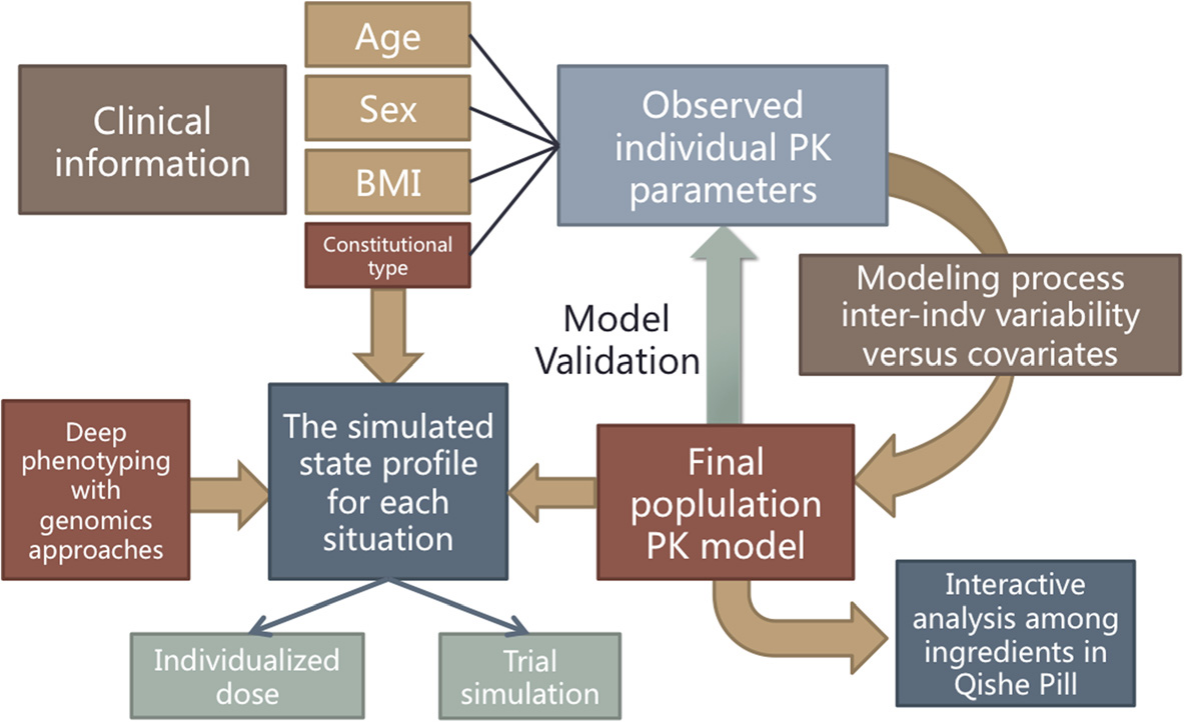
**Architecture and basic elements of the population pharmacokinetic (PopPK) research based on medical informatics and data management.**

For the final model, a backward elimination process will be employed to identify significant covariates. The covariates in the full model will be excluded one by one. The objective function value (OFV) will be compared with that of the full model. A covariate will be retained in the model when its elimination results in an increase in the OFV of 7.88 (chi-square, *P* <0.005, *df* = 1). We will select the model according to the reduction in the OFV value, goodness-of-fit plots, reduction in the IIV of structure model parameters, residual error, robust model parameter estimation, and model stability. The architecture and basic elements of the PopPK research is illustrated in Figure 2.

## Discussion

Cervical radiculopathy is a significant public health problem worldwide, accounting for 60 to 70% of all cervical spondylopathy cases; it is generally believed that most symptoms can be cured or relieved by nonsurgical treatments [25].

Previous evidence-based trials have reported that analgesics and nerve-nutrition medicines have considerable effects on neck pain. Even though analgesics, such as aspirin, seem to be relatively safe, gastrointestinal reactions are often reported after their use [26]. Mecobalamin, a neuropathy drug, which relieves neck pain by stimulating the inhibition of nerve degeneration and improving the excitability of nerve fibers [27], seems to be ineffective when degeneration occurs.

The Qishe pill provides specific benefits for “Qi and Blood”, terms in *TCM*, meaning that it can profit and activate blood circulation. We have previously shown that the Qishe pill reversibly inhibits the NF-κB pathway, which might be an underlying mechanism behind its anti-inflammatory potential in disc degeneration *in vivo* [28]. Extensive investigations have confirmed that the Qishe pill has several therapeutic features, inhibiting platelet surface activity and platelet aggregation [29], reducing the synthesis and release of local PGE [30], decreasing malondialdehyde content in inflammatory exudates [31], and inhibiting the IL-1beta-induced phosphorylation of extracellular signal-regulated kinases 1/2 and c-Jun N-terminal kinase [32]_._ These effects include predictable pharmacokinetics (PK) and pharmacodynamics (PD) behaviors; in addition, the Qishe pill suppresses acute inflammatory reactions and pain events with fewer gastrointestinal discomfort and rash. A simple and sensitive method using ultra-high-performance liquid chromatography–tandem mass spectrometry (UHPLC–MS/MS) on a reverse-phase C18 column was developed for the simultaneous determination of the 19 major components of the Qishe pill [20]. This method was successfully validated for the quantification of the 19 components in the Qishe pill products, and constitutes a new standard in quality assessment of TCM prescriptions containing multiple bioactive components (see Additional file 1). Over the last few years, several interactions between the Qishe pill ingredients and the pharmacokinetics of flavonoids (calycosin and calycosin-7-o--d-glycoside), alkaloids (tetrandrine, fangchinoline, magnoflorine and berberine hydrochloride), ferulic acid and glycosides (astragaloside IV, astragalussaponin I and III) have been reported [20,33-37]. Furthermore, HPLC-TQ/MS was employed to detect the component contents in the Qishe pill, whose partial pharmacokinetic parameters are shown in Table 5.

**Table 5:** **Pharmacokinetic parameter after oral administration of Qishe pill at a dose of 6.33 g•kg-1 to SD rats (mean ± SD, n = 6)**

**Parameters**	***SEA***	***SEI***	***FOR***	***ONO***	***5-O-M***	***AST-IV***
***t***_1/2_ **(h)**	22.72 ± 11.49	12.35 ± 10.3	51.24 ± 44.19	7.04 ± 3.7	42.79 ± 16.29	11.41 ± 12.51
***C***_max_ **(ng/ml)**	53.71 ± 16.14	136.51 ± 45.24	9.39 ± 4.32	2.45 ± 1.51	0.14 ± 0.07	13.3 ± 5.42
***T***_max_ **(h)**	0.22 ± 0.07	0.31 ± 0.16	0.22 ± 0.07	0.25 ± 0.00	0.25 ± 0.00	2.00 ± 2.25
***AUC***_last_(h*ng/ml)	606.87 ± 161.89	851.3 ± 251.31	38.1 ± 8.04	4.17 ± 1.53	1.13 ± 0.10	107.74 ± 65.65
***AUC***_tot_(h*ng/ml)	826.3 ± 229.51	900.76 ± 236.67	71.57 ± 26.49	4.89 ± 1.65	2.31 ± 0.41	129.75 ± 61.01
***MRT*** **(h)**	36.54 ± 15.27	16.58 ± 6.89	70.4 ± 60.63	9.55 ± 4.04	65.96 ± 20.60	17.9 ± 17.38
***CL*** **(L/h/kg)**	0.05 ± 0.01	0.02 ± 0.01	0.02 ± 0.00	0.12 ± 0.05	0.16 ± 0.03	0.06 ± 0.07
***V*** **z (L/kg)**	1.41 ± 0.67	0.41 ± 0.33	0.98 ± 0.43	1.12 ± 0.83	9.38 ± 2.09	0.83 ± 0.94

**Figure 3:**
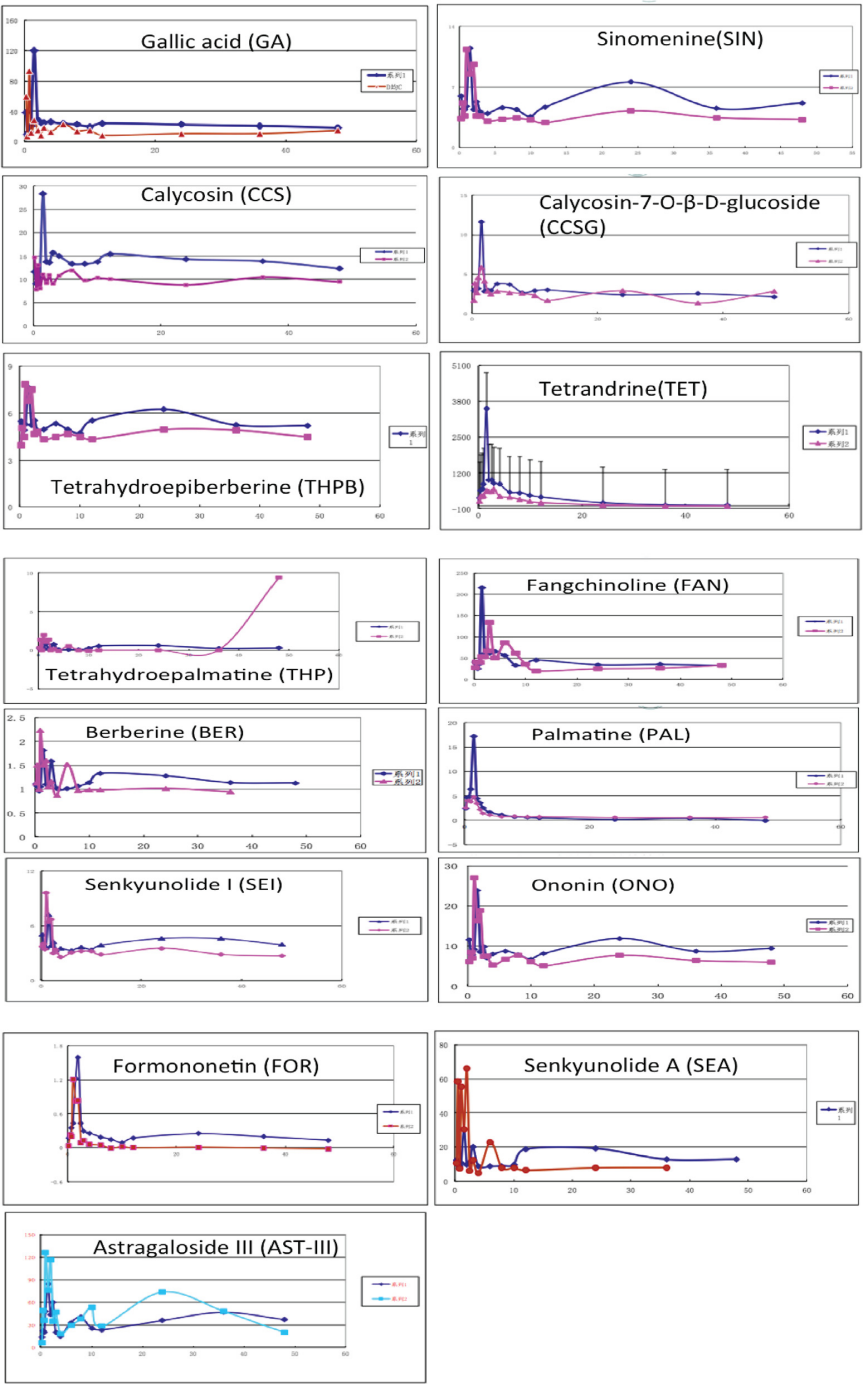
**Mean plasma concentration-time curves of seven components after oral administration of the Qishe pill at medium and high dosages (n = 4).**

In the pre-trial of limited size (n = 6), accordingly, a UPLC-TQ/MS method for simultaneous determination of plasma concentrations of 15 bio-active Qishe pill constituents was developed and validated. The results showed that the method was selective, sensitive, precise, accurate, and reliable for comparative quantification of ferulic acid, calycosin, calycosin glycosides,calycosin-7-O-β-glucoside, ononin, formononetin, astragaloside, tetrandrine, fangchinoline base, berberine, tetrahydroberberine, tetrahydropalmatine, sinomenine and magnolia base, in human plasma samples. The mean plasma concentration–time profiles of the major bio-active components determined after oral administration of Qishe pills are illustrated in Figure 3.

According to the results of the pilot trial, further research should be conducted with modified protocol in dosage setting, requirements of sample collection (diet specifications, blood-collection time window) and sample processing.

### Highlights

Diagnosis and treatment based on an overall analysis of the illness and patient’s condition is the essence of the *TCM* theory. Rational medication is dependent upon a basic understanding of the way patients handle drugs (pharmacokinetics) and their response to specific drug effects (pharmacodynamics). Moreover, *TCM* constitutional types in traditional Chinese recipes could significantly influence the plasma concentration-time and their pharmacokinetic parameters after oral administration [38,39]. As data collection and OMICS analyses in PopPK of the Qishe pill will be processed simultaneously, research is focusing on the analyses of PK, OMICS and laboratory data to generate new hypotheses for translational research such as individualized dose, individualized scope of *TCM* and trial simulation.

The present study will establish three cohorts to detect potential differences in pharmacokinetic characteristics of the Qishe pill constituents in the three different *TCM* constitutional types, to assess whether the rate and extent of drug metabolism will be altered in *Qi-*deficiency or *Blood*-stasis constitutional types. If the differences in the Qishe Pill pharmacokinetics among the three major constitutional groups were known, personalized medication strategies for people with the three major constitutional types could be provided for the application of TCM.

## Trial status

Participant recruitment and prescreening began in December 2014. Blank control serum samples have been collected for standardization of the general methodology and normal conditions.

## Additional file

Additional file 1:
**Typical chromatograms of ultrahigh-performance LC and tandem mass spectrometry (UHPLC-MS/MS) for (a) the mixed standard substance and (b) a sample [** 32 **].**

## Abbreviations

AST-III: Astragaloside III
AST-IV: Astragaloside IV
*AUC*: area under the plasma concentration–time curve
BER: Berberine
CA: Cholic acid
CCS: Calycosin
CCSG: Calycosin-7-O-β-D- glucoside
CE: Collision energy
*C*_max_: Maximum plasma concentration
DF: Distribution volume
CL/F: Apparent clearance
CL_M_: Clearance
DAI: Daidzein
FAN: Fangchinoline
FOCE: First-order conditional estimation methods
FOCE-I: First-order conditional estimation methods with interaction
FOR: Formononetin
GA: Gallic acid
*k*_e_: Elimination rate constant
MS: Mass spectrometry
MRM: Multiple reaction monitoring
NMR: Nuclear magnetic resonance
OFV: Objective function value
ONO: Ononin
PAL: Palmatine
PD: Pharmacodynamics
PK: Pharmacokinetics
PopPK: Population pharmacokinetic
SEA: Senkyunolide A
SEI: Senkyunolide I
SIN: Sinomenine
TCM: Traditional Chinese Medicine
*T*_max_: Time to maximum concentration
UHPLC–MS/MS: Ultra-high-performance liquid chromatography–tandem mass spectrometry
V/F: Apparent distribution volume
V_M_: Distribution volume
5-O-M: 5-O-methylvisammioside
