# Supplementary figures and images for: Population pharmacokinetic modeling of the Qishe pill in three major traditional Chinese medicine-defined constitutional types of healthy Chinese subjects: study protocol for a randomized controlled trial
Source: Trials. 2015 Feb 26;16:64. doi: 10.1186/s13063-015-0568-6 (PMC4351929; doi:10.1186/s13063-015-0568-6)

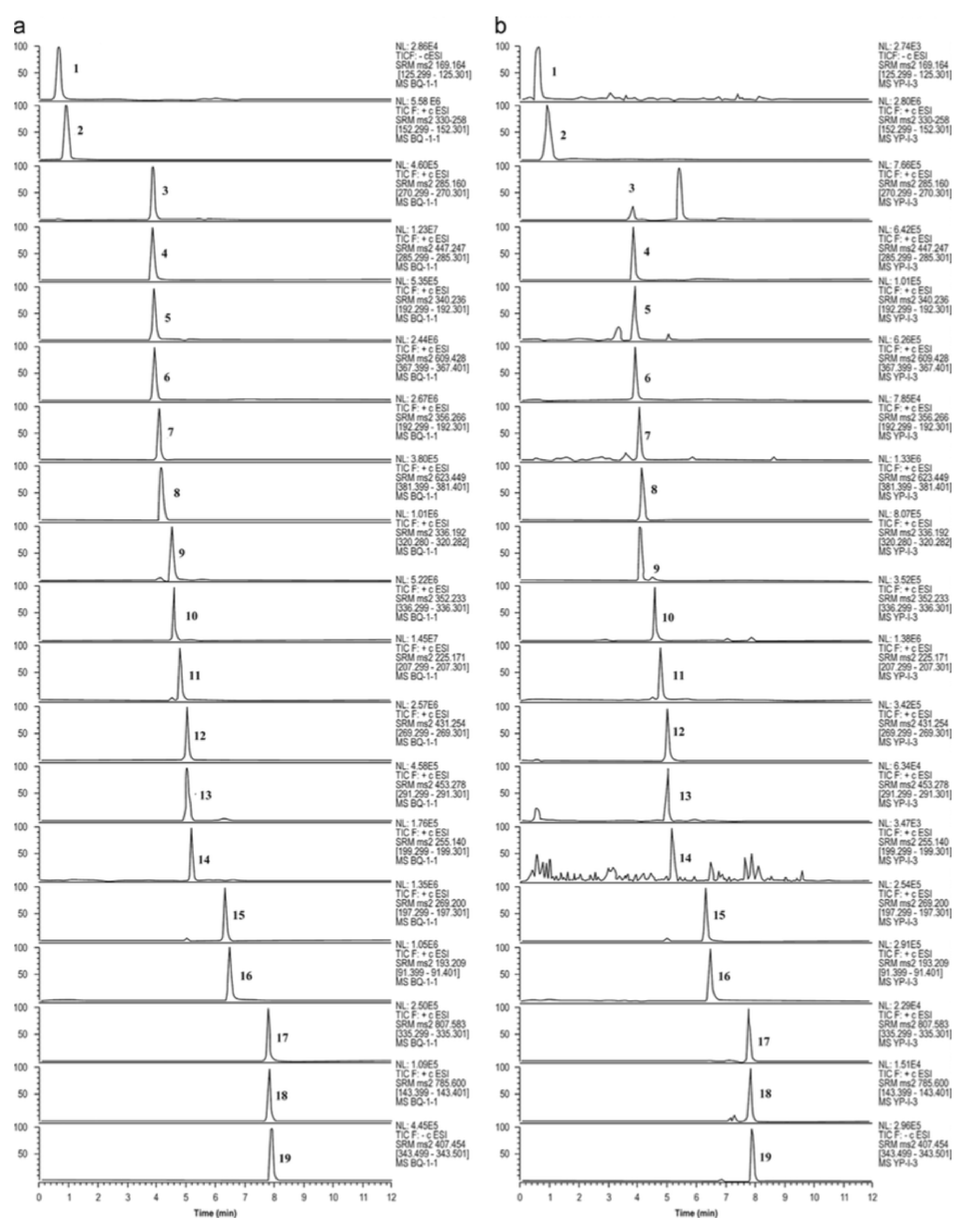

Supplement: Additional file 1: — Typical chromatograms of ultrahigh-performance LC and tandem mass spectrometry (UHPLC-MS/MS) for (a) the mixed standard substance and (b) a sample [ 32 ]. [file 13063_2015_568_MOESM1_ESM.jpeg]
